# Exosomal microRNA-140-3p from human umbilical cord mesenchymal stem cells attenuates joint injury of rats with rheumatoid arthritis by silencing SGK1

**DOI:** 10.1186/s10020-022-00451-2

**Published:** 2022-03-18

**Authors:** Yijiang Huang, Liang Chen, Daosen Chen, Pei Fan, Huachen Yu

**Affiliations:** 1 Department of Orthopaedic, The Second Affiliated Hospital and Yuying Children’s Hospital of Wenzhou Medical University, 109 West College Road, Wenzhou, 325000 China; 2 Key Laboratory of Orthopaedics of Zhejiang Province, Wenzhou, 325000 China

**Keywords:** Rheumatoid arthritis, MicroRNA-140-3p, Exosome, Serum-and glucocorticoid-inducible kinase 1, Joint injury, Rat

## Abstract

**Objective:**

Over the years, microRNAs (miRNAs) have been involved in the pathogenesis of rheumatoid arthritis (RA). We aim to investigate the role of human umbilical cord mesenchymal stem cells (HUCMSCs)-derived exosomal miR-140-3p in RA development.

**Methods:**

Exosomes(exo) were isolated from human umbilical cord-derived mesenchymal stem cells (HUCMSCs), and this isolation was followed by the transfer of miR-140-3p. RA rat models were constructed by collagen II adjuvant and respectively treated with HUCMSCs-exo or HUCMSCs-exo carrying miR-140-3p mimic/inhibitor, and expression of miR-140-3p and serum- and glucocorticoid-inducible kinase 1 (SGK1) was assessed. Then, RA score and inflammation scoring, fibrosis degree and apoptosis, serum inflammatory response and oxidative stress in joint tissues were determined. The RA synovial fibroblasts (RASFs) were extracted from rats and identified. Conducted with relative treatment, the migration, proliferation and apoptosis in RASFs were determined.

**Results:**

MiR-140-3p was decreased while SGK1 was increased in RA rats. HUCMSCs-exo or upregulated exosomal miR-140-3p improved pathological changes and suppressed inflammation, oxidative stress and fibrosis in RA rats, and also constrained and RASF growth. Overexpression of SGK1 reversed the inhibition of RASF growth caused by overexpression of miR-140-3p.

**Conclusion:**

Upregulated exosomal miR-140-3p attenuated joint injury of RA rats by silencing SGK1. This research provided further understanding of the role of exosomal miR-140-3p in RA development.

**Supplementary Information:**

The online version contains supplementary material available at 10.1186/s10020-022-00451-2.

## Introduction

Rheumatoid arthritis (RA) is a systemic autoimmune disease that initially occurs at joints. Chronic inflammatory processes results in bone erosions and cartilage damage, and then induces destruction of the total joint structure (Fatel et al. 2018). Featured by a symmetrical inflammatory polyarthritis, RA affects about 0.5–1.0% of the population (England et al. 2018), and continues to be the main health burden affecting life quality and healthcare resource consumption, especially in low- and middle-income countries, such as Asian developing countries (Lau et al. 2019). It has been found by etiological researches that RA is related to genetic and experimental factors, such as viral and bacterial infection, smoking, sex hormones, sunlight and vitamin D (Rajaei et al. 2019). Although the RA patients could be pharmacologically treated, many of them still have symptoms such as pain and fatigue, and RA medications also have side effects (Prothero et al. 2018). Thus, it is urgent to find potential biomarkers for RA administration.

Human umbilical cord mesenchymal stem cells (HUCMSCs) are an outstanding stem cell type used for allogenic cell-based therapy due to their advantageous ethical access, affluent tissue source, low immunogenicity and rapid renewal property (Yang et al. 2020a). It has been reported that HUCMSCs had an immunomodulatory effect on T lymphocytes in RA (Ma et al. 2019). Exosomes are small (diameter of 40–100 nm) membrane vesicles that released from multiple cell types (Sun et al. 2018). HUCMSCs-derived exosomes (HUCMSCs-exo) have all of the advantages of HUCMSCs and avoid their disadvantages. It is also easier to quantify HUCMSCs-exo and maintain their bioactivities in preservation and transport (Ding et al. 2018). HUCMSCs-exos have been demonstrated to inhibit apoptosis in disuse osteoporosis (Yang et al. 2020b), while their impact on RA remains unknown. Exosomes usually regulate the local intercellular communication through conveying coding RNAs, non-coding RNAs, antigen presentation molecules, DNA and proteins (Sun et al. 2018). MicroRNAs (miRNAs) are non-coding short-arm (18–22 base pair) RNAs participating in various biological processes via repressing the translation or degrading the transcription of target genes (Chen et al. 2015). Genetic variances of some miRNA genes have been reported to predispose an individual to RA development. For miR-140 variances, Liu et al. have depicted that miR-140-3p contributes to repressing cell viability and accelerating apoptosis of synovial fibroblasts in RA (Zu et al. 2021), while miR-140-3p depletion exerted the opposite effects on RA development (Zhong et al. 2020). Furthermore, various exosomal miRNAs were implicated in the pathophysiology of multiple autoimmune diseases. For instance, the depleted levels of exosomal miR-150-5p can induce exacerbated RA’s aggressive properties and angiogenesis (Chen et al. 2018). In addition, the exosomal miR-302d has recently been elucidated to attenuate the pathological consequences of patients with systemic lupus erythematosus (Wu et al. 2017). As for the exosomal miRNAs derived from mesenchymal stem cells, it has been disclosed that serum exosomal miR-146a can regulate mesenchymal stem cells senescence in patients with systemic lupus erythematosus (Dong et al. 2019). For the function of miR-140-3p in autoimmune diseases, it has been demonstrated that the abnormal expression of miR-140-3p in synovial fluid is related to osteoarthritis severity (Yin et al. 2017). Moreover, the downregulation of exosomal miR-140-3p has been confirmed in multiple myeloma patients (Zhang et al. 2019), while the role of HUCMSCs-derived exosomal miR-140-3p in RA has not been uncovered yet. Serum- and glucocorticoid-induced protein kinase 1 (SGK1) belongs to the SGK1 family and is under the transcriptional control of serum and glucocorticoids (Akhoon et al. 2019). It has been verified that SGK1 is an essential regulator of osteo-/chondrogenic transdifferentiation and calcification of vascular smooth muscle cells (Schelski et al. 2019). However, the effect of SGK1 on RA and the target relationship between miR-140-3p and SGK1 remain unexplored.

We aim to explore the impact of HUCMSCs-derived exosomal miR-140-3p on RA development by regulating SGK1. We hypothesized that the elevation of exosomal miR-140-3p may alleviate joint injury of RA rats via silencing SGK1.

## Materials and methods

### Ethics statement

Written informed consent was acquired from all patients before this study. The protocol of this study was confirmed by the Ethic Committee of The Second Affiliated Hospital and Yuying Children’s Hospital of Wenzhou Medical University. Animal experiments were strictly in accordance with the Guide to the Management and Use of Laboratory Animals issued by the National Institutes of Health. The protocol of animal experiments was approved by the Institutional Animal Care and Use Committee of The Second Affiliated Hospital and Yuying Children’s Hospital of Wenzhou Medical University.

### Isolated culture and identification of HUCMSCs

The human umbilical cords that obtained from healthy caesarean delivered uterogestation fetus from gynecology and obstetrics of The Second Affiliated Hospital and Yuying Children’s Hospital of Wenzhou Medical University were used for the extraction of HUCMSCs. It was sterile and microbial pollution-free during the surgery.

The umbilical cords were cut into 5 cm segments, which were washed by phosphate buffered saline (PBS) and centrifuged at 400×*g* for 6 min, and then were resuspended in Dulbecco’s modified Eagle medium (DMEM)/12F containing 12% fetal bovine serum (FBS) until the cell confluence reached 80%, and then were trypsinized and passaged to 10 cm culture dishes for multiplication culture (P1). The medium was replaced or cells were detached and subcultured every 3 or 4 days.

The morphology and adherence of HUCMSCs were observed under a microscope and P3 HUCMSCs were subpackaged into flow tubes (2 × 10^5^ cells/tube). The tubes were respectively supplemented with CD29-phycoerythrin (PE), CD44-PE, CD73-fluoresceine isothiocyanate (FITC), CD90-PE, CD105-PE, CD11b-PE, CD14-PE、CD19-activated protein C (APC), CD31-PE and human leukocyte antigen-antigen D related (HLA-DR)-PE antibodies for 30-min incubation at 4 °C with light avoidance. A flow cytometer (BD Biosciences, NJ, USA) was used for detection.

### Extraction and identification of exosomes

Exosomes were extracted using an exosome extraction kit. First, the supernatant of P3–P5 HUCMSCs was collected and added with ExoQuick TC exosome extraction reagent (SBI, USA) at 1:5 for overnight incubation at 4 °C (> 12 h). The supernatant was discarded and the sample was centrifuged at 1500×*g* for 5 min, and all the liquid was removed. Finally, the obtained exosomes were resuspended with 100–500 μL PBS. The morphology of exosomes was observed using a conventional transmission electron microscope (JEM-1200EX, Hitachi, Tokyo, Japan). The particle size distribution of exosomes was detected by NanoSight and the expression of exosome marker proteins (CD81 and Alix) was detected by Western blot analysis.

### Establishment of RA rat models

Male Wistar rats aged 6 week (Kay Biological Technology Co., Ltd., Shanghai, China) were seeded with 100 μL bovine collagen II (Chondrex, Redmond, WA, USA) and 100 μL complete Freund’s adjuvant added with mycobacterium tuberculosis (Biolead, Beijing, China). Twenty-one days later, a booster injection was given with the same dose of bovine collagen II and incomplete Freund’s adjuvant. After the second injection, when the limbs and feet of rats were obviously swelled, ankle diameter and claw volume were increased (Zheng et al. 2020).

The rats were divided into seven groups: the sham group (normal rats), the chronic infectious arthritis (CIA) group, the exo group (modeled rats were injected with exosomes), the mimic-negative control (NC)-exo group (modeled rats were injected with miR-140-3p mimic NC-transfected exosomes), the miR-140-3p mimic-exo group (modeled rats were injected with miR-140-3p mimic-transfected exosomes), the inhibitor-NC-exo group (modeled rats were injected with miR-140-3p inhibitor NC-transfected exosomes) and the miR-140-3p inhibitor-exo group (modeled rats were injected with miR-140-3p inhibitor-transfected exosomes). Rats in the treatment were injected with 100 µL exosomes or miRNA-transfected exosomes every week (1 µg/µL) (Chen et al. 2020).

### RA score

RA degree was scored on the 42nd day: 0–1 score: no detectable pathology (the appearance was normal with a flexible/evasive body, and the paw could support the bodyweight with highest grip strength); 1–2 scores: arthritis onset (slight swelling of the joint above the paw); 2–4 scores: mild arthritis (swelled joint with inflammation); 4–6 scores: mild to moderate arthritis (swelled joint with the last finger deformed inward, the paw could transiently support the bodyweight with decreased flexibility and grip strength); 6–8 scores: severe arthritis (severe swelling of joint, paw and finger, with deformed joints and legs, lack of support in the upper part, loss of weight, lack of flexibility and grip strength, climbing and eating were affected) (Hu et al. 2020).

### Preparation of joint tissue sections

Rats were euthanized by overdosage of anesthesia and the joint tissues were collected. The harvested joint tissues were fixed with formaldehyde for 24 h, decalcified using ethylene diamine tetraacetic acid, dehydrated by gradient ethanol and permeabilized by xylene. Embedded with paraffin, the tissues were cut into 6 μm median sagittal plane sections. Paraffin sections were used for hematoxylin–eosin (HE) staining, Masson staining and terminal deoxynucleotidyl transferase-mediated deoxyuridine triphosphate nick end-labeling (TUNEL) staining. Another joint tissues were taken and placed in a -80 refrigerator for reverse transcription quantitative polymerase chain reaction (RT-qPCR) and Western blot analysis.

### HE staining

The joint tissue sections of rat right postal paws were dewaxed with xylene and dehydrated by ethanol. After HE staining, the sections were sealed by neutral gum and the inflammatory infiltration in synovium, synovial hyperplasia and bone/cartilage destruction (Wang et al. 2020) were observed under a light microscope and scored. The inflammation scores ranked 0–4 points and the scoring method was referred by Camp et al. (2005).

### Masson staining

Paraffin sections were dewaxed to water and stained with Weigert iron hematoxylin for 5 min, differentiated using acid acetic acid differentiation solution, returned to blue using 0.7% Masson modified trichromatic dye (sigma-Aldrich, CA, USA), and stained with ponceau fuchsin staining solution for 6 min, and then were dehydrated, permeabilized and sealed. The changes in articular cartilage were evaluated by observing the staining of collagen fibers under a light microscope. According to the Ashcroft score, the fibrosis status was quantified (Huang et al. 2020b).

### TUNEL staining

The TUNEL kits were obtained from Keygen Biotech Co., Ltd. (Nanjing, China) and used for evaluating the apoptosis. The sections were dewaxed, hydrated, treated with 3% H_2_O_2_ for 10 min and incubated with terminal deoxynucleotidyl transferase and digoxigenin-11-uridine-5ʹ-triphosphate incubation buffer for 1.5 h. Then, sections were developed with diaminobenzidine, slightly counterstained by hematoxylin and observed under a microscope. Cells with nuclei had brownish-yellow particles were positive cells, namely apoptotic cells. The results were judged by the blind method combined with a CIAS-1000 image analysis system software. Under a light microscope. Five fields of view were randomly selected for the counting of positive cells.

### Culture of RA synovial fibroblasts (RASFs)

Primary RASFs were isolated from CIA rat synovial tissues by 1.5 mg/mL collagenase and 0.04% hyaluronidase, and the cells were cultured in DMEM containing 10% FBS, 100 U/mL penicillin and 100 μg/mL streptomycin. P3 RASFs were used as the experimental cells.

### Identification of RASFs

An inverted microscope was used to observe the RASFs: P3 cells rapidly grew and were homogeneous, fusiform and plump in a tumor-like growth. These cells were RASFs and had highly expressed vimentin. The P3 cells were trypsinized and cell concentration was adjusted to 1 × 10^6^ cells/mL by DMEM. Seeded onto 24-well plates at 10^5^ cells/well, the cells were incubated for 12 h, fixed with ethanol, permeabilized by Triton X-100, sealed by blocking buffer for 30 min and incubated with primary antibody vimentin at 4 °C overnight and fluorescent secondary antibodies at 4 °C for 1 h. A fluorescence microscope was used for observation.

### Cell grouping

The RASFs were classified into the following groups: the blank group (normally cultured RASFs), the exo group (RASFs and HUMSCs-exo were co-cultured for 3 d), the mimic-NC-exo group (RASFs and HUMSCs-exo that had been transfected with miR-140-3p mimic NC were co-cultured for 3 days), the miR-140-3p mimic-exo group (RASFs and HUMSCs-exo that had been transfected with miR-140-3p mimic were co-cultured for 3 days), the mimic-NC group (RASFs were transfected with miR-140-3p mimic NC), the miR-140-3p mimic group (RASFs were transfected with miR-140-3p mimic) and the miR-140-3p mimic + oe-SGK1 group (RASFs were transfected with miR-140-3p mimic and SGK1 overexpressed vector).

### 3-(4,5-dimethyl-2-thiazolyl)-2,5-diphenyl-2-H-tetrazolium bromide (MTT) assay

Cell viability was measured with the MTT assay. Briefly, cells in each well were incubated with 5 mg/mL MTT solution (20 µL/well) at 24 h, 48 h and 72 h of the transfection at 37 °C for 4 h. The medium was removed and each well was appended with 150 µL dimethyl sulfoxide. An absorbance (A) at 490 nm was assessed by a microplate reader (Anthos Labtec Instruments GmbH, Austria) until the crystal dissolved. The experiment was repeated 3 times and the cell growth curves were graphed.

### Transwell assay

Cells were collected to prepare cell suspension with the cell density of 5 × 10^5^ cells/mL. Cells suspension (200 µL) was seeded into Transwell apical chambers and the basolateral chambers were added with 700 µL DMEM containing 20% FBS. After 12-h incubation, cells on the surface of apical chambers were removed and cells in the basolateral chambers were stained by hematoxylin for 2 min. The number of transmembrane cells was counted in five fields of view under a microscope.

### Flow cytometry

Based on the instructions of apoptosis detection kits (Keygen Biotech), cells from each treatment group were counted and 5 × 10^5^ cells in each well were reacted with a mixture of 50 µL binding buffer and 5 µL 7-amino-actinomycin D for 15 min without light exposure. Subsequently, the cells were successively supplemented with 450 µL binding buffer and 1 µL Annexin V-PE for 15-min reaction with light avoidance, and then detected by a flow cytometer.

### Enzyme-linked immunosorbent assay (ELISA)

According to protocols of ELISA kits (Vafioskan Flash; Thermo Fisher Scientific, MA, USA), the contents of tumor necrosis factor-α (TNF-α), interleukin (IL)-1β, superoxide dismutase (SOD) and malondialdehyde (MDA) in rat serum and cells were determined.

### RT-qPCR

Trizol kits (Invitrogen Inc., CA, USA) were used to extract the total RNA, and the RNA was reversely transcribed into cDNA using Mir-X miRNA First-Strand Synthesis kit (Takara, Dalian, China) and PrimeScript RT reagent kit (Takara). RT-qPCR was performed using a CFX Connect Real-Time PCR Detection System (Bio-Rad Laboratories, Singapore). Glyceraldehyde phosphate dehydrogenase (GAPDH) and U6 were respectively used as the internal reference of SGK1 and miR-140-3p. Data were analyzed by 2^−ΔΔCt^ method. All the primers for genes in this study are shown in Additional file 1: Table S1.

### Western blot analysis

Tissues, cells and exosomes were lysed in radioimmunoprecipitation assay (RIPA) buffer (SolarBio) containing the protease inhibitor cocktail (Merck, Darmstadt, Germany). The protein concentration was determined with a BCA kit (Beyotime Biotechnology) according to the protocol. The samples (20 μg) were boiled with 5× sodium dodecyl sulfate (SDS) at 99 °C in a metal bath for 15 min, centrifuged at 12,000×*g* for 5 min, loaded and conducted with 10% SDS-polyacrylamide gel electrophoresis. Blocked with 5% skim milk for 1 h, the membranes were incubated with primary antibodies SGK1 (1:1000, Abcam, MA, USA), CD81 (1:800), Alix (1:500) and GAPDH (1:1000) (Santa Cruz Biotechnology Inc., CA, USA) at 4 °C overnight, and then incubated with relative secondary antibody for 1.5 h. Enhanced chemiluminescent reagent (Millipore Inc., MA, USA) was used for development and a chemiluminescence gel imaging system was utilized for imaging and photographing.

### Dual luciferase reporter gene assay

Binding sites of miR-140-3p and SGK1 were predicted by a bioinformatic software, and wild type (WT) and mutant (MUT) SGK1 3’untranslated region (3’UTR) with miR-140-3p binding sites were amplified by PCR. The amplification products were conducted with gel electrophoresis and gel recycling of the target gene, and then inserted with Xba I and Fse I sites of pGL3 luciferase report plasmid (Promega Corporation, WI, USA) to construct pGL3-SGK1-3ʹ-UTR and PSGK1-3ʹ-UTR-MUT vectors. RASFs were transfected with miR-140-3p mimic and mimic-NC based on the directions of lipofection transfection. The luciferase activity was determined by a detector.

### Statistical analysis

All data analyses were conducted using GraphPad Prism Version 6.0 (GraphPad Software, La Jolla, USA) and SPSS 21.0 software (IBM Corp. Armonk, NY, USA). Data were expressed as mean ± standard deviation. The t-test was performed for comparisons between two groups, analysis of variance (ANOVA) was used for comparisons among multiple groups and Tukey’s post hoc test was used for pairwise comparisons after ANOVA. *P* value < 0.05 was indicative of statistically significant difference.

## Results

### Identification of HUCMSCs, HUCMSCs-exo and RASFs

**Fig. 1:**
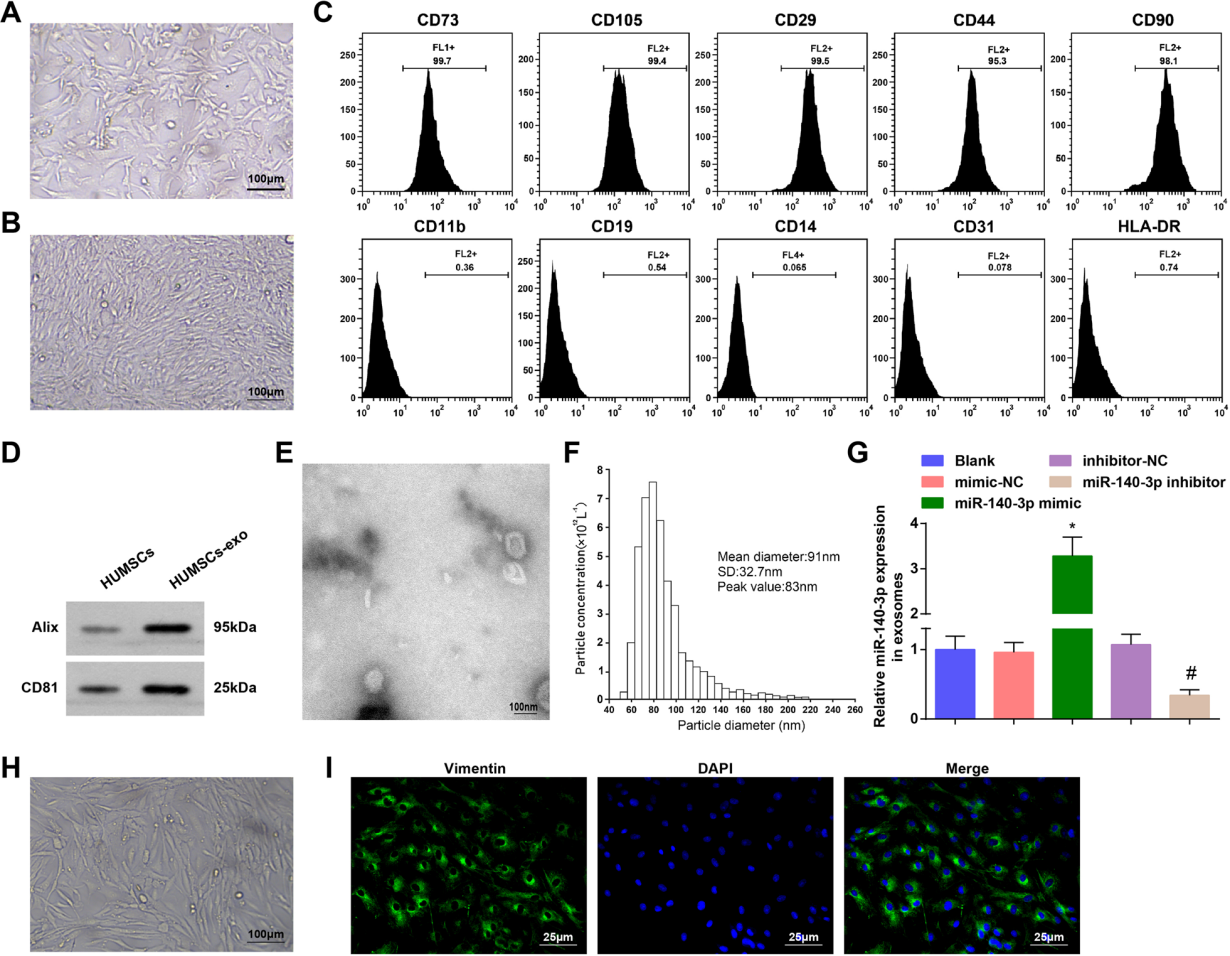
Identification of HUCMSCs, HUCMSCs-exo and RASFs. **A** Morphological observation of low density HUMSCs; **B** Morphological observation of high density HUMSCs ; **C** Surface antigens of HUMSCs were detected by flow cytometry; **D** Expression of CD81 and Alix in exosomes was determined by Western blot analysis; **E** Exosomes were identified through a TEM; **F** Particle size of exosomes was analyzed by NTA; **G** Expression of exosomal miR-140-3p was assessed by RT-qPCR; **H** Morphological observation of rat RASFs ; **I** Rat RASFs were identified by vimentin protein immunofluorescent staining; N = 3; *P < 0.05 *vs* the mimic-NC group, ^#^*P* < 0.05 vs the inhibitor-NC group; the data were expressed as mean ± standard deviation, ANOVA was used for comparisons among multiple groups and Tukey’s post hoc test was used for pairwise comparisons after ANOVA

HUMSCs belong to the MSCs and have self-renewal property, multi-differentiation potential, anti-inflammation capacity, immunomodulatory effect and paracrine character (Cai et al. 2020). The morphology of HUCMSCs was observed under a microscope and it was found that cells were long fusiform and adhered well (Fig. 1A); the cells were in a whorl-like arrangement when there was a larger cell density (Fig. 1B). A flow cytometer was used to determine the cell surface markers and we found that CD29, CD44, CD73, CD90 and CD105 were positively expressed while CD11b, CD14, CD19, CD31 and HLA-DR were negatively expressed in the cells (Fig. 1C), which were in accordance with the reported criterion of MSCs (Lv et al. 2014).

The common markers of exosomes (CD81 and Alix) were detected using Western blot analysis. CD81 is a tetraspanin and Alix is a protein participating in vasculogenesis. Both of the proteins were enriched in exosomes from different sources. Our results indicated that CD81 and Alix expressed in HUCMSCs-exo, but nearly did not express in HUCMSCs (Fig. 1D). The exosome morphology was observed by a TEM and it was found that the exosomes had a vesicle-like structure (Fig. 1E). We observed in NTA that the particle size of HUCMSCs-exo was (91.0 ± 32.7) nm, in line with the essential characteristics of exosomes (Zaborowski et al. 2015) (Fig. 1F). These data suggested that the HUCMSCs-exos were successfully extracted.

MiR-140-3p expression in exosomes was gauged after HUCMSCs were transfected with miR-140-3p mimic or inhibitor. It came out that in exosomes, miR-140-3p mimic elevated miR-140-3p expression while miR-140-3p inhibitor suppressed miR-140-3p expression (Fig. 1G).

Rat RASFs were observed and we discovered that there were abundant cells with large volume, plump morphology and a rapid proliferation rate (Fig. 1H). It was found through vimentin protein immunofluorescent staining that the vimentin proteins of most of the RASFs were positively expressed and performed as green fluorescence (Fig. 1I), indicating the rat RASFs were successfully obtained.

### HUCMSCs-exo or exosomal miR-140-3p attenuates joint injury of RA rats and reduced miR-140-3p reverses the effect of HUCMSCs-exo on joint injury of RA rats

Exosomes derived from MSCs have immunomodulatory properties of MSCs. It has been reported that HUCMSCs-exo can promote cartilage regeneration in a full-thickness cartilage defect model (Toh et al. 2017). In addition, a study has shown that the expression of miR-140-3p is related to RA (Ntoumou et al. 2017).

**Fig. 2:**
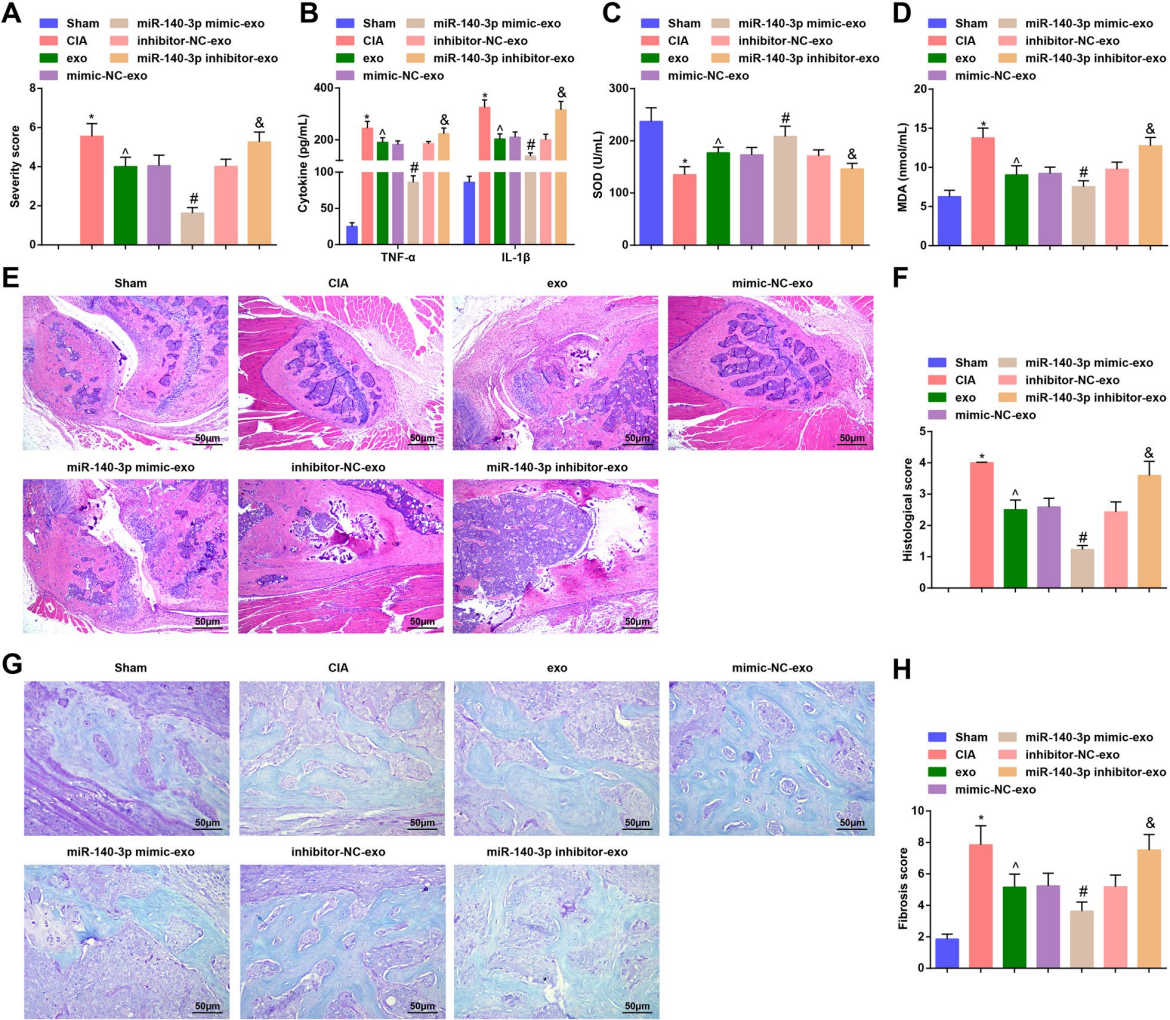
HUCMSCs-exo or exosomal miR-140-3p attenuates joint injury of RA rats and reduced miR-140-3p reverses the effect of HUCMSCs-exo on joint injury of RA rats. **A** Pathological changes in rat feet; **B** Serum contents of TNF-α and IL-1β in rats; **C** Serum activity of SOD in rats; **D** Serum content of MDA in rats; **E** Pathology of rat ankle joints was observed through HE staining; **F** RA score of rats after HE staining; **G** Masson staining was used to observe the fibrosis in tibial articular cartilage of rats; **H** Fibrosis was quantified based on Masson staining; n = 5; *P < 0.05 vs the sham group, ^*P* < 0.05 vs the CIA group, ^#^*P* < 0.05 vs the mimic-NC-exo group, ^&^*P* < 0.05 vs the inhibitor-NC-exo group; the data were expressed as mean ± standard deviation, ANOVA was used for comparisons among multiple groups and Tukey’s post hoc test was used for pairwise comparisons after ANOVA

To explore the therapeutic effect of exosomes and miR-140-3p on RA, we established a rat model of CIA and injected the CIA rats with exosomes. The results of RA score indicated that relative to the sham-operated rats, the foot pathology of CIA rats was aggravated; rats that had been treated with HUCMSCs-exo or upregulation of exosomal miR-140-3p had a decreased degree of foot pathology; the foot pathology of rats treated with exosomes inhibiting miR-140-3p was exacerbated (Fig. 2A).

Serum levels of inflammatory factors and oxidative stress indicators in rats were measured by ELISA. The outcomes suggested that CIA rats had higher contents of TNF-α, IL-1β and MDA, and a lower level of SOD activity in relation to the sham-operated ones; treatment of HUCMSCs-exo or upregulation of exosomal miR-140-3p suppressed contents of TNF-α, IL-1β and MDA, and promoted SOD activity; the contents of TNF-α, IL-1β and MDA were enhanced while SOD activity was repressed by exosomes inhibiting miR-140-3p (Fig. 2B–D).

HE staining revealed that sham-operated rats had normal structures of the right postal paw. In the CIA rats and CIA rats treated with exosomes reducing miR-140-3p, there were obvious inflammatory cell infiltration in the joint cavity, lymphocyte infiltration in synovial tissues, synoviocyte hyperplasia and inflammatory cell infiltration and erosion in bones. In CIA rats treated with HUCMSCs-exos and exosome conveying mimic-NC or inhibitor-NC, there were partial synovial hyperplasia and bone destruction; the joint structure of rats in the miR-140-3p mimic-exo group was improved versus the CIA group and there were attenuated inflammatory cell infiltration and slight synoviocyte hyperplasia were stilled observed and the symptoms were abated (Fig. 2E). Rats were scored under a microscope and we found that the CIA rats had higher points than the sham-operated ones, while the points were decreased after the treatment of HUCMSCs-exo or elevation of exosomal miR-140-3p. Exosomes downregulating miR-140-3p inhibitor resulted in higher points (Fig. 2F).

The fibrosis degree in rat tibial joint tissues was observed through Masson staining and it was observed that in the sham-operated rats, the stroma and collagen fibers were blue, stained evenly, and gradually deepened towards the calcification layer. The tidal line was clear, the boundary between cartilage and subchondral bone was clear, the dense bone and trabecular bone of subchondral bone were blue, and bone collagen was red. In CIA rats and CIA rats treated with exosomes inhibiting miR-140-3p, the results showed that the surface of articular cartilage was rough and missing, the surface cartilage was denatured and unstained, the fibrosis was obvious, a large number of red-stained flame-like processes existed near the tidal line, bone trabeculae was unstained, and the boundary between cartilage and subchondral bone was not clear. The fibrosis degree was attenuated in CIA rats treated with exosomes and exosomes transmitting mimic-NC or inhibitor-NC. In CIA rats treated with exosomes elevating miR-140-3p, the surface of articular cartilage was smooth and there was no obvious degeneration, unstained and fibrosis (Fig. 2G). The fibrosis  score evaluation suggested that CIA rats had higher OARSI scores, while were decreased by exosomes or exosomes upregulating miR-140-3p. Exosomes downregulating miR-140-3p treatment led to increased fibrosis score (Fig. 2H).

These findings implied that exosomes and elevated exosomal miR-140-3p relieve the joint injury in RA rats, while this effect could be reversed by inhibiting miR-140-3p.

### HUCMSCs-exo or exosomal miR-140-3p alleviates chondrocyte apoptosis in RA rats and decreased miR-140-3p reverses the impact of HUCMSCs-exo on chondrocyte apoptosis in RA rats

**Fig. 3:**
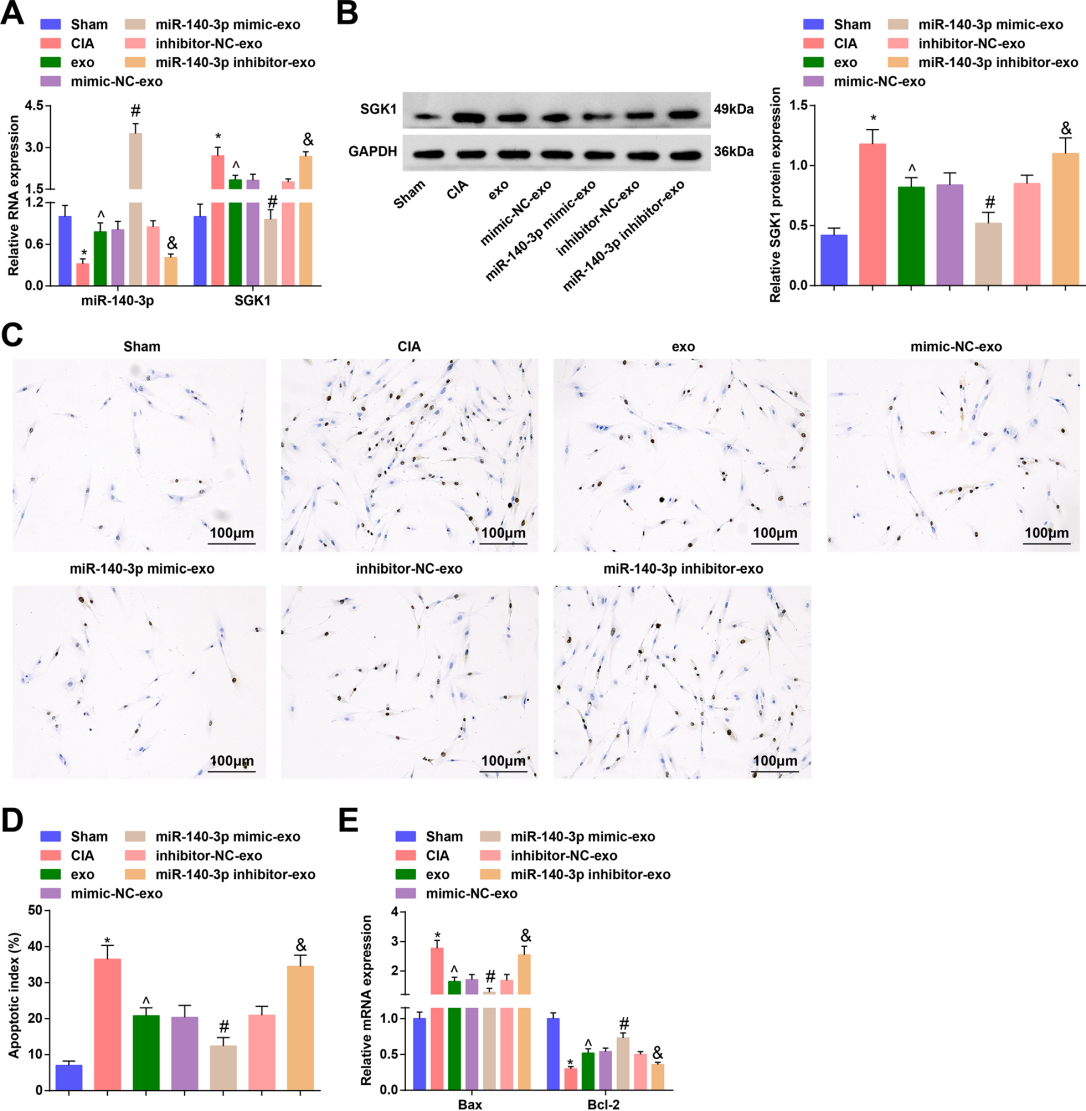
HUCMSCs-exo or exosomal miR-140-3p alleviates chondrocyte apoptosis in RA rats and decreased miR-140-3p reverses impact of HUCMSCs-exo on chondrocyte apoptosis in RA rats. **A** Expression of miR-140-3p and SGK1 in cartilage tissues was determined by RT-qPCR; **B** Protein expression of SGK1 in cartilage tissues was determined by Western blot analysis; **C** Apoptosis of chondrocytes was observed through TUNEL staining; **D** AI of chondrocytes in each group; **E** mRNA expression of Bax and Bcl-2 in cartilage tissues was determined using RT-qPCR; n = 5; *P < 0.05 vs the sham group, ^*P* < 0.05 vs the CIA group, ^#^*P* < 0.05 vs the mimic-NC-exo group, ^&^*P* < 0.05 vs the inhibitor-NC-exo group; the data were expressed as mean ± standard deviation, ANOVA was used for comparisons among multiple groups and Tukey’s post hoc test was used for pairwise comparisons after ANOVA

We further detected miR-140-3p and SGK1 expression using RT-qPCR and Western blot analysis. It came out that miR-140-3p expression was decreased while SGK1 expression was increased in CIA rats. The exosomes or exosomes elevating miR-140-3p upregulated miR-140-3p and downregulated SGK1 (Fig. 3A, B).

Results of TUNEL staining and RT-qPCR analysis revealed that apoptotic index (AI) and Bax mRNA expression were increased while Bcl-2 mRNA expression was decreased in CIA rats. Treatment of HUCMSCs-exo or elevated exosomal miR-140-3p restricted AI and Bax mRNA expression while promoted Bcl-2 mRNA expression. CIA rats treated with inhibited exosomal miR-140-3p had higher AI and Bax mRNA expression and lower Bcl-2 mRNA expression (Fig. 3C–E).

The above data implied that HUCMSCs-exo or upregulated exosomal miR-140-3p inhibited chondrocyte apoptosis in RA rats, while decreased miR-140-3p reverses the impact of HUCMSCs-exo on chondrocyte apoptosis in RA rats.

### HUCMSCs-exo or exosomal miR-140-3p decelerates proliferation and accelerates apoptosis of RASFs

**Fig. 4:**
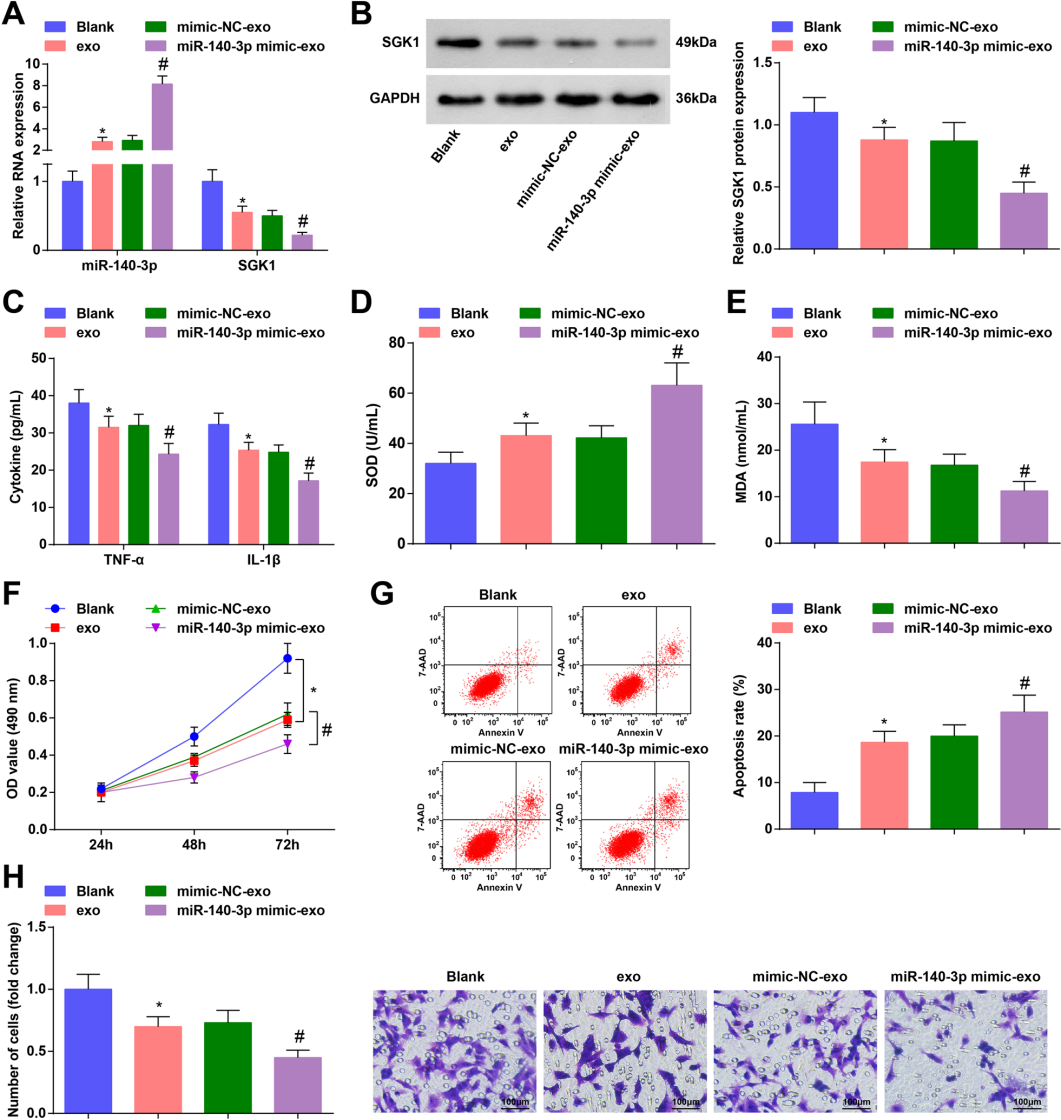
HUCMSCs-exo or exosomal miR-140-3p decelerates proliferation and accelerates apoptosis of RASFs. **A** Expression of miR-140-3p and SGK1 in RASFs was detected by RT-qPCR; **B** Protein expression of SGK1 in RASFs was measured using Western blot analysis; **C** Contents of TNF-α and IL-1β in RASFs were evaluated by ELISA; **D** SOD activity in RASFs was measured by ELISA; **E** MDA content in RASFs was measured by ELISA; **F** MTT assay was applied to assess the proliferation of RASFs; **G** Apoptosis of RASFs was detected by flow cytometry; **H** Migration ability of RASFs was evaluated by Transwell assay; N = 3; *P < 0.05 vs the blank group, ^#^*P* < 0.05 vs the mimic-NC-exo group; the data were expressed as mean ± standard deviation, ANOVA was used for comparisons among multiple groups and Tukey’s post hoc test was used for pairwise comparisons after ANOVA

Next, RASFs were extracted and co-cultured with HUCMSCs-exo or exosomes transmitting mimic-NC or miR-140-3p mimic. Outcomes of RT-qPCR and Western blot analysis indicated that miR-140-3p was upregulated but SGK1 was downregulated after treatment of HUCMSCs-exo, while exosomes upregulating miR-140-3p increased miR-140-3p expression and decreased SGK1 expression (Fig. 4A, B).

Contents of TNF-α, IL-1β, MDA and SOD in RAFSs were determined by ELISA, and we discovered that HUCMSCs-exo or upregulated exosomal miR-140-3p suppressed the contents of TNF-α, IL-1β and MDA, whereas elevated SOD activity (Fig. 4C–E).

MTT assay was performed to assess the proliferation of RAFSs. Results reflected that RAFSs co-cultured with HUCMSCs-exo had a lower proliferation level. The RAFS proliferation was further repressed by exosomes upregulating miR-140-3p (Fig. 4F).

Apoptosis of RASFs was measured using flow cytometry and we found that RASFs that had been treated with HUCMSCs-exo or elevation of exosomal miR-140-3p had a higher apoptosis rate (Fig. 4G).

Transwell assay was applied to assess the migration ability of RASFs and the outcomes implied that the treatment of HUCMSCs-exo or upregulated exosomal miR-140-3p restricted the migration ability of RASFs (Fig. 4H).

Our results showed that HUCMSCs-exo or exosomal miR-140-3p repressed proliferation and promoted apoptosis of RASFs.

### MiR-104-3p targets SGK1

**Fig. 5:**
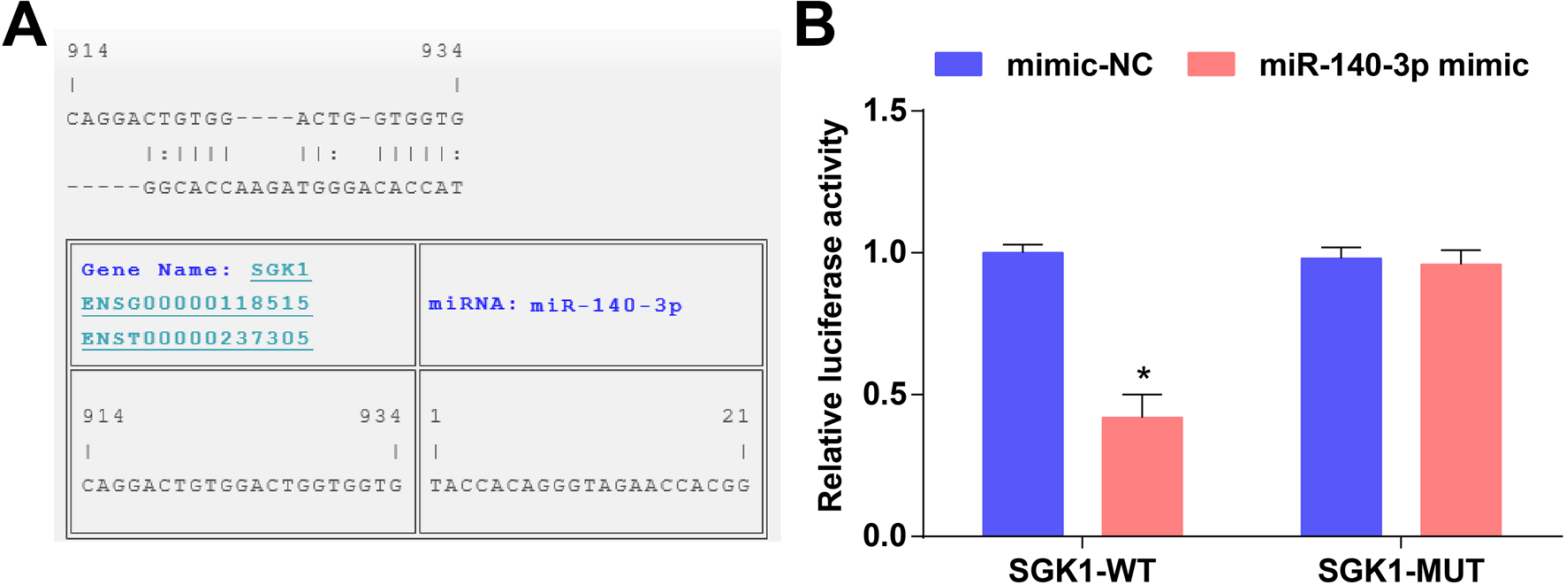
MiR-140-3p targets SGK1. **A** Binding sites of miR-140-3p and SGK1 were predicted by a bioinformatic website; **B** Target relationship between miR-140-3p and SGK1 was confirmed by dual luciferase report gene assay; *P < 0.05 vs the mimic-NC group; the data were expressed as mean ± standard deviation and the t-test was performed for comparisons between two groups

It was predicted by a bioinformatic website that there existed binding sites of miR-140-3p and SGK1 (Fig. 5A). The luciferase activity of RASFs was repressed after the co-transfection of miR-140-3p mimic and SGK1-WT plasmid; the co-transfection of miR-140-3p mimic and SGK1-MUT plasmid didn’t affect the luciferase activity, indicating a targeting relationship between miR-140-3p and SGK1 (Fig. 5B).

### Elevated miR-140-3p restrains proliferation and facilitates apoptosis of RASFs and this effect can be reversed by overexpressed SGK1

**Fig. 6:**
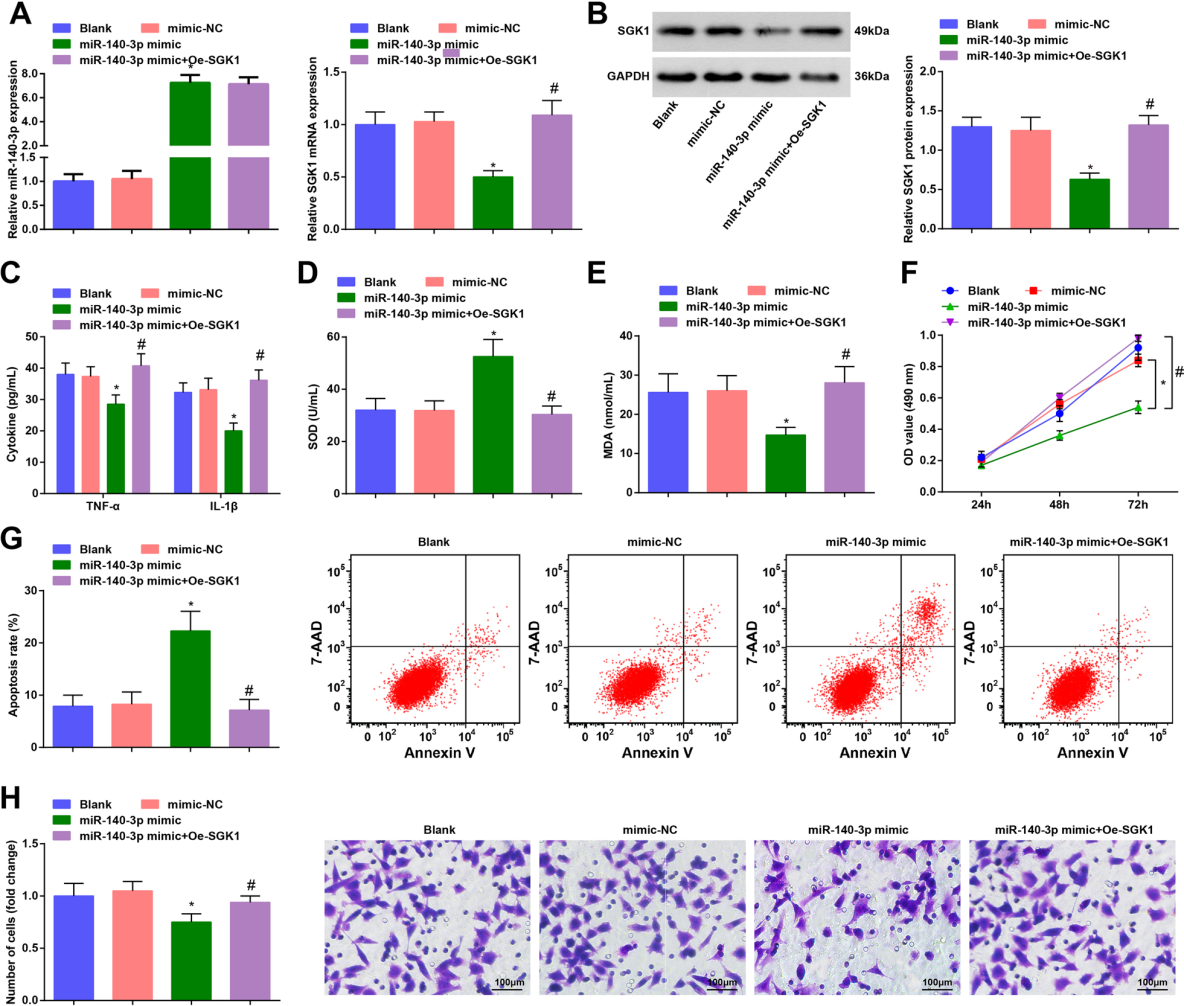
Elevated miR-140-3p restrains proliferation and facilitates apoptosis of RASFs and this effect can be reversed by overexpressed SGK1. **A** Expression of miR-140-3p and SGK1 in RASFs was detected by RT-qPCR; **B** Protein expression of SGK1 in RASFs was measured using Western blot analysis; **C** Contents of TNF-α and IL-1β in RASFs were evaluated by ELISA; **D** SOD activity in RASFs was measured by ELISA; **E** MDA content in RASFs was measured by ELISA; **F** MTT assay was applied to assess the proliferation of RASFs; **G** Apoptosis of RASFs was detected by flow cytometry; **H** Migration ability of RASFs was evaluated by Transwell assay; N = 3; *P < 0.05 vs the mimic-NC group, ^#^*P* < 0.05 vs the miR-140-3p mimic group; the data were expressed as mean ± standard deviation, one-way ANOVA was used for comparisons among multiple groups and Tukey’s post hoc test was used for pairwise comparisons after ANOVA

Subsequently, we further studied the effect of miR-140-3p targeting SGK1 on RA. MiR-140-3p and SGK1 expression in RASFs was assessed and it was found that miR-140-3p upregulation increased miR-140-3p expression while repressed SGK1 expression, and the downregulation of SGK1 that induced by miR-140-3p elevation was reversed by upregulated SGK1 (Fig. 6A, B).

As evaluated by ELISA, contents of TNF-α, IL-1β and MDA was reduced while SOD activity was elevated by miR-140-3p elevation, while these effects of miR-140-3p elevation were reversed by SGK1 upregulation (Fig. 6C–E).

The proliferation, apoptosis and migration of RASFs were determined by MTT assay, flow cytometry and Transwell assay, respectively. We discovered that miR-140-3p upregulation repressed proliferation and migration and promoted apoptosis of RASFs, and the effects of miR-140-3p mimic on proliferation, migration and apoptosis of RASFs were reversed by upregulated SGK1 (Fig. 6F–H).

These results suggested that miR-140-3p upregulation suppressed RASF growth and this effect could be abolished by SGK1 upregulation.

## Discussion

In recent years, accumulating evidence has suggested that the epigenetic mechanisms of miRNAs have been widely explored, which greatly contributed to RA pathogenesis (Evangelatos et al. 2019). MiRNAs are believed to be the most promising candidate biomarkers among various exosomal cargo molecules because of their high abundance, inherent stability, easiness of sampling and significance as global cellular regulators. The effect of exosomal miRNAs has received wide attention (Hu et al. 2019). We performed this research to explore the role of HUCMSCs-derived exosomal miR-140-3p in joint injury in RA, and we discovered that the upregulation of exosomal miR-140-3p relieved joint injury in RA rats by inhibiting SGK1.

We assessed miR-140-3p and SGK1 expression in rat cartilage tissues using RT-qPCR and/or Western blot analysis. The results indicated that miR-140-3p was lowly expressed while SGK1 was highly expressed in cartilage tissues of RA rats when compared with cartilage tissues from normal rats. In accordance with this finding, Yin et al. have demonstrated that miR-140-3p expression is reduced in osteoarthritis synovial fluid in relation to synovial fluid from non-osteoarthritis patients (Yin et al. 2017), and it has been revealed that the expression of miR-140-3p in exosomes of multiple myeloma patients is suppressed in comparison to healthy controls (Zhang et al. 2019). A recent research has clarified that SGK1 is overexpressed in osteoarthritis cartilage (Huang et al. 2020a). MiR-140-3p expression in cartilage tissues from rats that had been treated with HUCMSCs-exo was also determined and we found that the exosomes were able to upregulate miR-140-3p in RA rats. Consistently, Asano et al. (2017) have unraveled that exosomal miR-140-3p is broadly amplified in chronic myeloid leukemia patients with musculoskeletal pain relative to those without that pain. We also confirmed the target relationship between miR-140-3p and SGK1 using bioinformatic method and dual luciferase report gene assay, while this relation remains seldom explored.

Gain- and loss-of-function assays were used to assess the roles of altered miR-140-3p and SGK1 in the development of RA, and one of the results of both animal and cellular experiments indicated that HUCMSCs-exo, exosomal miR-140-3p or silenced SGK1 are able to restrain the inflammation response and oxidative stress in RA. In line with this result, Sun et al. (2018) have mentioned that HUCMSCs-exo attenuates inflammation in mice with spinal cord injury, and HUCMSCs-exos have been reported to decrease oxidative stress in liver injury mouse models and liver cells (Jiang et al. 2018). Moreover, Al-Modawi et al. (2019) have discovered that miR-140-3p inhibits inflammation and oxidative stress in human chondrocytes and osteoarthritis models, and it also has been identified that miR-140-5p alleviates oxidative stress in cisplatin-induced acute kidney injury (Liao et al. 2017). Furthermore, a previous study has revealed that SGK1 upregulates the inflammatory transcription factor nuclear factor-κB, thus stimulating the expression of diverse inflammatory mediators in tissue fibrosis (Artunc and Lang 2014). The in vivo experiments also implied that the apoptosis of chondrocytes in RA rats is decelerated by HUCMSCs-exo, exosomal miR-140-3p or silenced SGK1. Similarly, Chen et al. have figured out that HUCMSCs-exo represses bone tissue apoptosis in rats with necrosis of the femoral head (Li et al. 2019), and a recent document has unearthed that the upregulation of miR-140-3p reduces IL-1β-induced apoptosis of CHON-001 chondrocyte cells in osteoarthritis (Ren et al. 2020). In addition, our research suggested that HUCMSCs-exo, exosomal miR-140-3p or silenced SGK1 has the ability to restrain proliferation and promote apoptosis of RASFs. Consistent with this finding, HUCMSCs-exos have been verified to ameliorate liver (Li et al. 2013) and cardiac fibrosis (Zhao 2015). Li et al. (2017) have demonstrated that miR-140-5p constrains the proliferation of RASFs, and it has been reported that SGK1 induces proliferation of cardiac fibroblasts (Gan et al. 2018).

## Conclusion

Our study uncovered that the elevation of HUCMSCs-derived exosomal miR-140-3p plays a protective role against joint injury in RA through downregulating SGK1, and our findings may contribute to exploring potential biomarkers for RA treatment. However, the further molecular mechanisms were not fully studied here due to limited time and fund, and we would perform corresponding research in our future work.

## Supplementary Information

**Additional file 1: Table S1. **Primer sequences for PCR assay.

## Data Availability

Not applicable.

## Abbreviations

miRNAs: MicroRNAs
RA: Rheumatoid arthritis
HUCMSCs: Human umbilical cord mesenchymal stem cells
SGK1: Serum- and glucocorticoid-inducible kinase 1
RASFs: RA synovial fibroblasts
HUCMSCs-exo: HUCMSCs-derived exosomes
miRNAs: MicroRNAs
HLA-DR: Human leukocyte antigen-antigen D related
CIA: Chronic infectious arthritis
NC: Mimic-negative control
RT-qPCR: Reverse transcription quantitative polymerase chain reaction
HE: Hematoxylin–eosin
TUNEL: Terminal deoxynucleotidyl transferase-mediated deoxyuridine triphosphate nick end-labeling
RASFs: RA synovial fibroblasts
oe: Overexpressed
ELISA: Enzyme-linked immunosorbent assay
TNF-α: Tumor necrosis factor-α
IL: Interleukin
SOD: Superoxide dismutase
MDA: Malondialdehyde
GAPDH: Glyceraldehyde phosphate dehydrogenase
SDS: Sodium dodecyl sulfate
WT: Wild type
MUT: Mutant type
ANOVA: Analysis of variance
