# Supplementary material for: Exosomal microRNA-140-3p from human umbilical cord mesenchymal stem cells attenuates joint injury of rats with rheumatoid arthritis by silencing SGK1
Source: Mol Med. 2022 Mar 18;28:36. doi: 10.1186/s10020-022-00451-2 (PMC8932126; doi:10.1186/s10020-022-00451-2)
Supplement: Supplementary file 1 — Additional file 1: Table S1. Primer sequences for PCR assay. [file 10020_2022_451_MOESM1_ESM.docx]

**Supplementary Table 1** Primer sequences for PCR assay

| Gene | Primer sequence (5’–3’) |
| --- | --- |
| miR-140-3p | Forward: 5′-TACCACAGGGTAGAACCACGG-3′ |
| SGK1 | Forward: 5′-GGGTGCCAAGGATGACTTTA-3′ |
|  | Reverse: 5′-AACGATGTTTAGGGAGTGCAGATA-3′ |
| Bax | Forward: 5′-TTCATCGAGCCCAGCA-3′ |
|  | Reverse: 5′-CTCGCTCAGCTTCTTGGTC-3′ |
| Bcl-2 | Forward: 5′-CTGCACCTGACGCCCTTCACC-3′ |
|  | Reverse: 5′-CACATGACCCCACCGAACTCAAAGA-3′ |
| U6 | Forward: 5′-GCTTCGGCAGCACATATACTAAAAT-3′ |
|  | Forward: 5′-CGCTTCACGAATTTGCGTGTCAT-3′ |
| GAPDH | Forward: 5′-CAAGGTCATCCATGACAACTTTG-3′ |
|  | Reverse: 5′-GTCCACCACCCTGTTGCTGTAG-3′ |

Note: miR-140-3p, microRNA-140-3p; SGK1, serum- and glucocorticoid-inducible kinase 1; GAPDH, glyceraldehyde phosphate dehydrogenase.
